# Course of *Plasmodium* infection studied using 2D-COS on human erythrocytes

**DOI:** 10.1186/s12936-023-04611-5

**Published:** 2023-06-20

**Authors:** Malwina Birczyńska-Zych, Jacek Czepiel, Maria Łabanowska, Martyna Kucharska, Magdalena Kurdziel, Grażyna Biesiada, Aleksander Garlicki, Aleksandra Wesełucha-Birczyńska

**Affiliations:** 1 Department of Infectious and Tropical Diseases, Jagiellonian University, Medical College, Jakubowskiego 2, 30-688 Kraków, Poland; 2 Department of Infectious Diseases, The University Hospital in Kraków, Jakubowskiego 2, 30-688 Kraków, Poland; 3 Faculty of Chemistry, Jagiellonian University, Gronostajowa 2, 30-387 Kraków, Poland

**Keywords:** Malaria, *P. falciparum*, *P. vivax*, Human red blood cells, Raman microspectroscopy, EPR, 2D-COS, 2T2D

## Abstract

**Background:**

The threat of malaria is still present in the world. Recognizing the type of parasite is important in determining a treatment plan. The golden routine involves microscopic diagnostics of Giemsa-stained thin blood smears, however, alternative methods are also constantly being sought, in order to gain an additional insight into the course of the disease. Spectroscopic methods, e.g., Raman spectroscopy, are becoming increasingly popular, due to the non-destructive nature of these techniques.

**Methods:**

The study included patients hospitalized for malaria caused by *Plasmodium falciparum* or *Plasmodium vivax,* in the Department of Infectious Diseases at the University Hospital in Krakow, Poland, as well as healthy volunteers. The aim of this study was to assess the possibility of using Raman spectroscopy and 2D correlation (2D-COS) spectroscopy in understanding the structural changes in erythrocytes depending on the type of attacking parasite. EPR spectroscopy and two-trace two-dimensional (2T2D) correlation was also used to examine the specificity of paramagnetic centres found in the infected human blood.

**Results:**

Two-dimensional (2D) correlation spectroscopy facilitates the identification of the hidden relationship, allowing for the discrimination of Raman spectra obtained during the course of disease in human red blood cells, infected by *P. falciparum* or *P. vivax*. Synchronous cross-peaks indicate the processes taking place inside the erythrocyte during the export of the parasite protein towards the cell membrane. In contrast, moieties that generate asynchronous 2D cross-peaks are characteristic of the respective ligand-receptor domains. These changes observed during the course of the infection, have different dynamics for *P. falciparum* and *P. vivax*, as indicated by the asynchronous correlation cross-peaks. Two-trace two-dimensional (2T2D) spectroscopy, applied to EPR spectra of blood at the beginning of the infection, showed differences between *P. falciparum* and *P. vivax*.

**Conclusions:**

A unique feature of 2D-COS is the ability to discriminate the collected Raman and EPR spectra. The changes observed during the course of a malaria infection have different dynamics for *P. falciparum* and *P. vivax*, indicated by the reverse sequence of events. For each type of parasite, a specific recycling process for iron was observed in the infected blood.

**Graphical Abstract:**

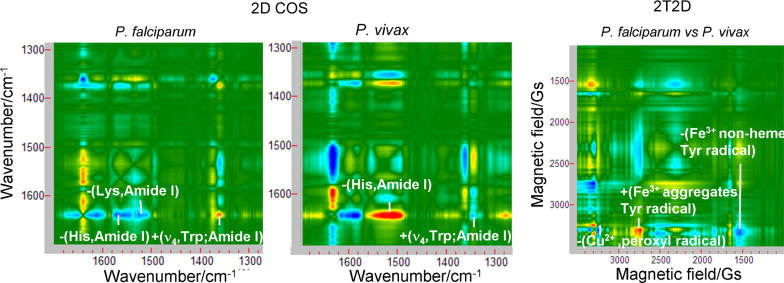

## Background

Malaria is one of the most dangerous infectious diseases [1]. Currently, it occurs in Poland as a disease imported from endemic regions [2]. Malaria is a mosquito-borne vector disease, caused by five species of parasite that are pathogenic to humans. Most malaria cases and deaths are thought to be caused by the *Plasmodium falciparum* infection [1]. Recently, however, it has been identified that the *Plasmodium vivax* infection and its consequences may have been underestimated and are, therefore, also life-threatening [3].

Malaria is a blood disease due to the tissue in which it develops. The parasites attack red blood cells, but not at random.* Plasmodium falciparum* can invade all erythrocyte subsets, while *P. vivax* preferentially invade immature red blood cells or, reticulocytes [4]. The life cycle of parasites in humans in the pre-erythrocytic stage is slightly different; it is 10 days and 12 days, respectively, for *P. falciparum* and *P. vivax*. Additionally, the dormancy of *P. vivax* hypnozoites varies over time, depending on the geographic location [5]. The 48-h development cycle in the host cell for both types of *Plasmodium* is similar; this includes around 20 h as a ring stage, then approximately 20 h as a trophozoite stage and finally around eight hours as a schizont. Parasites slightly differentiate the intra-erythrocyte development of gametocytes; in the case of *P. falciparum*, this process takes 15 days, and for *P. vivax* only 4 days [6, 7]. Anyone with a fever, returning from an endemic area, should have malaria in the differential diagnosis. The gold standard of diagnostics is the parasitological examination of Giemsa-stained capillary blood.

The aim of this study was to assess the possibility of using Raman spectroscopy in understanding certain structural changes in erythrocytes, depending on the type of the attacking parasite. The work to date on distinguishing the effect of parasites on blood cells using statistical methods has been promising [8]. 2D correlation analysis was also chosen because it was successful in understanding the aging process of healthy red blood cells and identifying the specific aging of cells infected with *P. falciparum* [9–11]. Moreover, in the presented studies, EPR measurements were made to check whether it is possible to identify infected blood according to the species of *Plasmodium* and by using magnetic methods. Identifying the disease-causing species is of clinical importance, therefore, it is important in diagnostics.

## Methods

### Blood samples

The spectroscopic measurements were carried out on blood samples collected from patients at the Department of Infectious Diseases, University Hospital in Kraków, Poland, who had been diagnosed with malaria caused by *P. falciparum* or *P. vivax*, as well as healthy volunteers. The blood of five patients from each infected group, with comparable parasitaemia, was taken for Raman and EPR analysis.

The blood from patients diagnosed with malaria was obtained by venipuncture. The blood was immediately transported to the Raman laboratory in specialized tubes containing heparin as an anticoagulant. A smear was then made, but to measure it, the cells were allowed to stabilize for approximately 10 min. Raman spectroscopic measurements were performed on the first, fifth and tenth day of hospitalization; the last day was characterized by negative parasitological test results.

### Statistical analysis of standard laboratory tests

In order to check whether there are statistically significant differences in standard medical tests between two groups of malaria patients, the Mann–Whitney *U* test was used. The following descriptive statistics were included in the statistical analysis: mean (M), standard deviation (SD), minimum (Min), maximum (Max), median (Me), first (Q1) and third quartile (Q3). A statistical analysis was performed using Statistics 25 software (IBM SPSS, USA). The threshold of statistical significance included values of p < 0.05.

### Raman microspectroscopy

A Renishaw InVia spectrometer, working in a confocal mode, connected to a Leica optical microscope with a 100× magnification objective (NA = 0.9) and an argon laser line of 514.5 nm were used to measure single erythrocytes. The laser power was kept low, ca. 1–3 mW at the sample, to ensure minimum invasion into the cells. The averaged spectra were made in Thermo Scientific Omnic v. 9.3 software.

### EPR spectroscopy

EPR measurements were performed using an X-band Bruker ELEXSYS 500 spectrometer (Karlsruhe, Germany) with 100 kHz field modulation. The samples of *P. falciparum* and *P. vivax*-infected whole blood, collected at the beginning of hospitalization, as well as the blood of healthy volunteers, were sealed in EPR tubes and the spectra were recorded at 77 K with a modulation amplitude of 0.5 mT.

### 2D-COS analysis

Generalized 2D correlation analysis was performed using the Noda’s method [12, 13]. The averaged spectra for all of the measured erythrocytes of 10 patients (five infected with *P. falciparum* and five with *P. vivax*) on the first, fifth and tenth day of hospitalization were considered as so-called dynamic spectra. The time of hospitalization was regarded as an external perturbation. The analysed spectra were pre-processed: smoothed, baseline corrected and normalized with the Renishaw WiRE 3.4 and 2.0 software and subjected to 2D correlation analysis.

EPR spectra of the *Plasmodium* infected at the beginning of the infection process and healthy infected whole blood constituted an input data for generating 2T2D correlation maps [14]. The software package for the calculation and presentation of the 2D spectra is built into the Win-IR Pro (Bio-Rad) software.

## Results and discussion

### The results of statistical analysis of standard laboratory tests

The analysis concerns the comparison of two groups of patients diagnosed with malaria species, 65.6% of cases (n = 21) were *P. falciparum* and 21.9% (n = 7) were *P. vivax*. No statistically significant differences were observed between the two groups of patients, see Table 1.

**Table 1:** Descriptive statistics on the analyzed parameters in a group of patients diagnosed with *P. vivax* and *P. falciparum* malaria (The Mann–Whitney *U* test)

Parameter	Malaria species	M	SD	Min.	Max.	Q1	Me	Q3	Statistical test result
WBC [10^3^/μl] max	*P. vivax*	6.95	1.53	4.59	9.29	5.83	7.19	7.83	U = 52; p = 0.25
	P. falciparum	6.39	2.72	2.5	15.6	4.82	6.31	6.98	
HGB [g/dl]	P. vivax	11.94	1.33	9.2	13.4	11.7	12.1	12.7	U = 63; p = 0.58
	P. falciparum	11.2	2.23	6.9	14.3	9.75	10.9	13.25	
PLT [10^3^/μl]	*P. vivax*	116.71	89.67	43	261	65	72	232	U = 55.5; p = 0.34
	*P. falciparum*	98.05	117.28	10	537	23	52	128,5	
ALT [U/l]	*P. vivax*	466.29	1102.72	18	2966	19	46	102	U = 63.5; p = 0.6
	*P. falciparum*	124.24	130.94	14	490	36,5	64	180,5	
Age	*P. vivax*	38.71	13.1	23	59	31	33	54	U = 70; p = 0.85
	*P. falciparum*	38.48	11.95	22	63	28	40	49	
CRP [mg/l]	*P. vivax*	68.11	55.34	1.13	128.54	2.83	79.68	119.3	U = 53; p = 0.56
	*P. falciparum*	84	70.43	3.89	263.88	18.91	84.7	120.01	
PCT [ng/ml]	*P. vivax*	12.47	17.59	0.06	32.6	0.06	4.74	–	U = 20; p = 0.77
	*P. falciparum*	5.47	7.38	0.15	25.83	0.54	2.2	8.14	
Parasitemia [%]	*P. vivax*	1.02	1.16	0.2	3	0.21	0.72	1.98	U = 49; p = 0.95
	P. *falciparum*	3.35	6.44	0.001	27.55	0.04	0.66	4.12	
AST	*P. vivax*	129.67	176.38	18	333	18	38	–	U = 13; p = 0.47
	*P. falciparum*	152	170.6	24	578	34.25	79	210.5	
ALP	*P. vivax*	77.27	22.12	64	102.8	64	65	–	U = 12; p = 0.78
	*P. falciparum*	81.38	33.51	41	133	54.5	67	117.51	
INR	*P. vivax*	1.15	0.21	0.88	1.45	0.97	1.11	1.35	U = 50.5; p = 0.9
	*P. falciparum*	1.15	0.14	0.99	1.44	1.04	1.12	1.21	
APTT	*P. vivax*	35.22	7.74	28	46.2	29.33	33.35	43	U = 38; p = 0.77
	*P. falciparum*	36	10.31	13.3	63.1	29.7	34.6	41.15	
Creatinine	*P. vivax*	90.14	18.43	58	108	74	100	104	U = 58; p = 0.41
	*P. falciparum*	83.29	21.37	49	121	65.1	85	98.35	
WBC [10^3^/μl] min	*P. vivax*	4.14	0.75	3.13	5.29	3.62	3.9	4.92	U = 39; p = 0.07
	*P. falciparum*	3.73	2.88	1.55	15.6	2.45	3.01	4.15	

### The Raman microspectroscopy of malaria-infected single human RBC

**Fig. 1:**
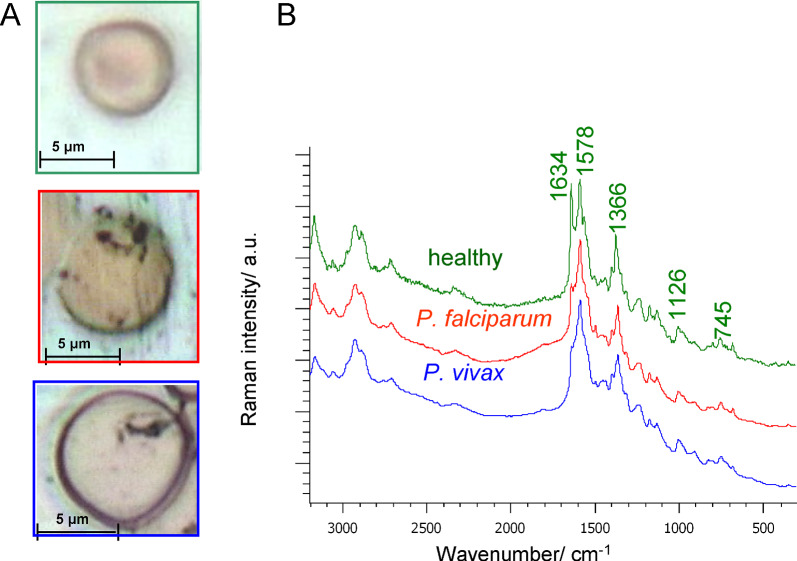
**A** microphotographs of the erythrocytes of healthy volunteers and malaria-infected erythrocytes with *P. falciparum* and *P. vivax* and with trophozoites in the form of a ring; **B** Raman spectra showing the most prominent peaks of the erythrocytes of healthy volunteers, infected with *P. falciparum* and *P. vivax*, in the range of 3200–300 cm^−1^. The values of the bands for healthy blood cells are marked. Excitation laser line at 514.5 nm; 100× magnification objective (NA = 0.90)

**Fig. 2:**
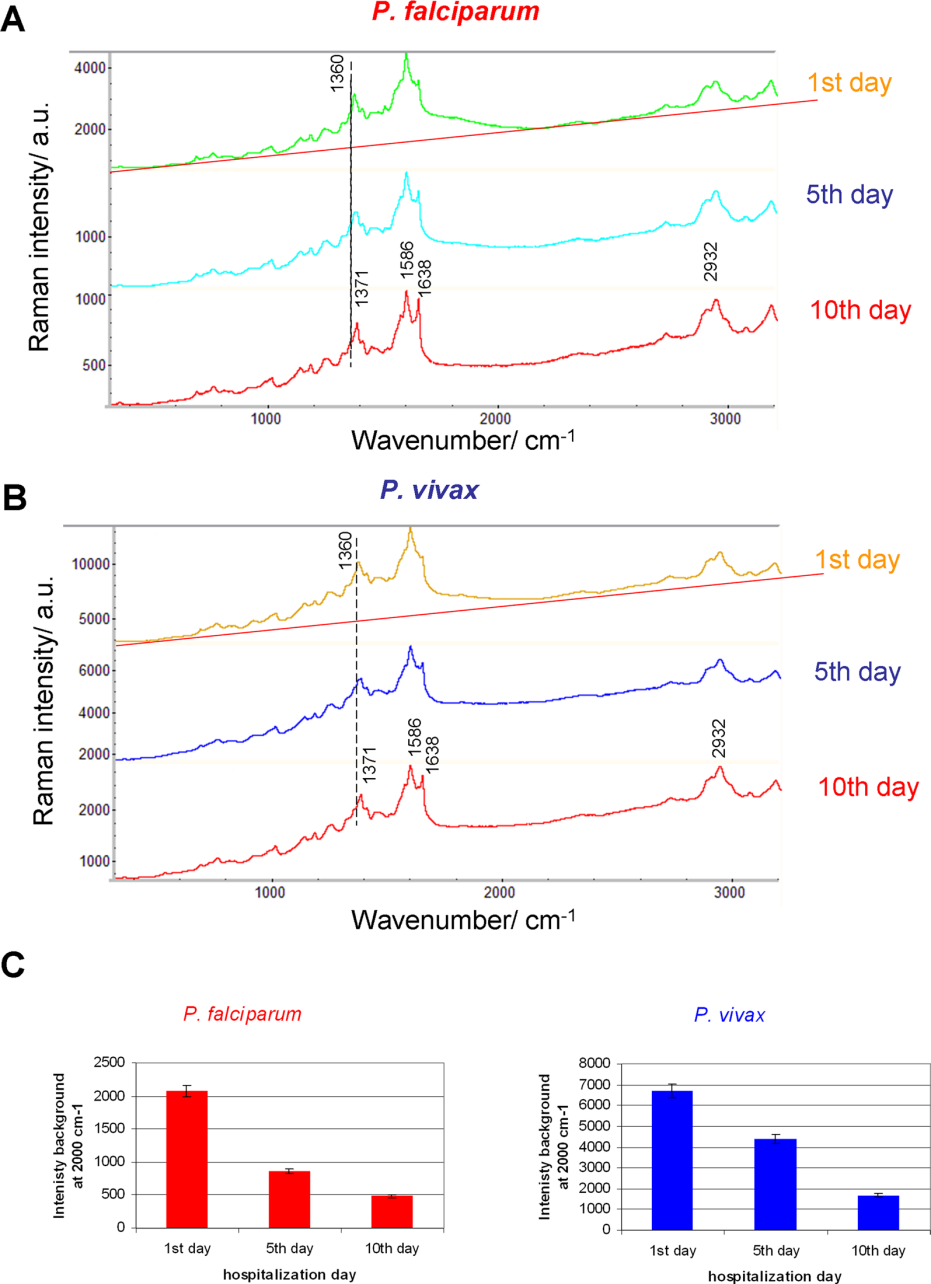
Averaged Raman spectra of all erythrocytes of five patients in each group, on the first, fifth and tenth day of hospitalization, **A** *P. falciparum*, **B** *P. vivax*, **C** the background intensity is determined for the wavenumber of 2000 cm^−1^ assuming a linear background, *vide* (**A**) and (**B**)

The activity of parasites changes the size and shape of red blood cells in a characteristic way. One of the observed features is the loss of the biconcave shape of the blood cell, see Fig. 1A. The blood of patients from malaria-infected groups was characterized by comparable parasitaemia. *P. falciparum* and *P. vivax* parasitaemia was 3.2 (0.27%) and 3.0 (0.2%), respectively. Raman spectroscopy enables the monitoring of haem modification taking place in the intra-erythrocytic phase of the *Plasmodium* life cycle [15]. The most characteristic is the shift of the ν_4_ band in the course of hospitalization from approximately 1360 cm^−1^ on the first day of hospitalization to 1373 cm^−1^ and 1371 cm^−1^ on the last day of hospitalization for *P. falciparum* and *P. vivax*, respectively (see Table 2). There is also a slight shift of haem ν_37_ vibration from positions 1586 cm^−1^ and 1585 cm^−1^ to 1587 cm^−1^ and 1586 cm^−1^ in the course of hospitalization for *P. falciparum* and *P. vivax*, respectively. In the case of both species, there is also a clearly visible increase in the ν_10_ band over the course of recovery. However, despite these minimal differences, the spectra of *Plasmodium-*infected red blood cells, at the same stage of the disease, are quite similar, see Figs. 1B, 2A and B. Due to this, it is difficult to determine the differentiating features, related to a specific species of parasite, directly from the spectra of erythrocytes. The noticeably higher background intensity observed in the spectra during the course of infection for *P. vivax* compared to *P. falciparum* is noteworthy, see Fig. 2C. The background intensity was determined for the wavenumber of 2000 cm^−1^ assuming a linear background course, as indicated in Fig. 2A and B. The elevated background appears to be a signal of inflammation in the body. It transpired that the background for the three analysed time periods in the course of infection is higher for *P. vivax*, see Fig. 2C [16]. The inflammatory response is related to the activation of signaling pathways that increase inflammation and the electron density in tissues and blood cells [17, 18]. Inflammation appears to last longer and recurs in the months following infection in *P. vivax* [19].

**Table 2:** Observed major Raman bands [cm^−1^] and their assignments for single human RBC: healthy, *P. falciparum* and *P. vivax* infected excited using 514.5 nm laser line

Healthy	Pf 1st day (av. 80 spectra)/10th day (av. 83 spectra)	Pv 1st day (av. 52 spectra)/10th day (av. 45 spectra)	Assignment	Local coordinates/description	References
2930	2929/2932	2931/2932	ν_sym_(CH_3_); PC	symmetrical CH stretching vibration of CH_3_	[8-10, 23]
1639	1638/1639	1637/1638	haem; ν_10_; amide I (conf. α)	ν(C_α_C_m_)_as_ ν(C = O), ν(CN) and δ(NH)	[8-10, 15, 23]
1586	1586/1587	1585/1586	haem; ν_37_; Asp; His;	ν(C_α_C_m_)_as_; phenyl mode ν _as_(COO^−^); ν(C_4_ = C_5_),	[8-10, 15, 22, 24, 28]
1372	1359/1372	1360/1371	haem; ν_4_; Trp	ν(C_α_C_m_)_as_ W7	[8-10, 15, 22, 28]
1170	1171/1170	1170/1169	haem (Hz); ν_30;_ Asp, Glu; Tyr; ν_9a_	_pyr_(half-ring)_as_ ν(CO); ν(CH); δ(COH)	[8-10, 15, 22, 23, 34]
1125	1128/1127	1127/1126	haem; ν_22;_ Asp, Glu;	ν_pyr _(half-ring)_as;_ ν(CO);	[8-10, 15, 22, 36]
1002	1001/1002	1000/1001	haem (Hz); ν_45_; Phe	ν(C_β_ -methyl); ν(CC), ring breathing;	[8-10, 15, 22, 23]
745	746/751	745/753	haem; ν_15_ Thr	ν(pyr. breathing) γ_rock_(CH_2_)	[8-10, 15, 23, 31]

*Pf* *P. falciparum*, *Pv* *P. vivax,* *Hz* haemozoin, *Asp* Aspartic acid, *Gln* Glutamine, *His* Histidine, *Phe* Phenylalanine, *Trp* Tryptophan, *Thr* Threonine, *Tyr* Tyrosine, *PC* phosphatidylcholine

### 2D correlation of malaria-infected single human RBC, infected with *Plasmodium*

Due to the similarity of the spectra of *Plasmodium-* infected red blood cells, at the same stage of the disease, by different species of *Plasmodium*, 2D correlation analysis (2D-COS) was performed to find hidden relationships. Construction of the two-dimensional spectrum (2D-COS) uses time-dependent fluctuations of spectroscopic signals, that change during treatment [12]. 2D-COS correlation spectroscopy is particularly helpful when disturbances in the studied systems are characterized by low variability, i.e. low amplitude. The 2D spectra obtained by this method, consisting of two types of dependency, synchronous and asynchronous, can highlight useful information often buried in the original time-resolved spectra. They are expressed in terms of the real and imaginary components of the Fourier transform of the dynamic spectrum.

### 2D synchronous correlation

**Fig. 3:**
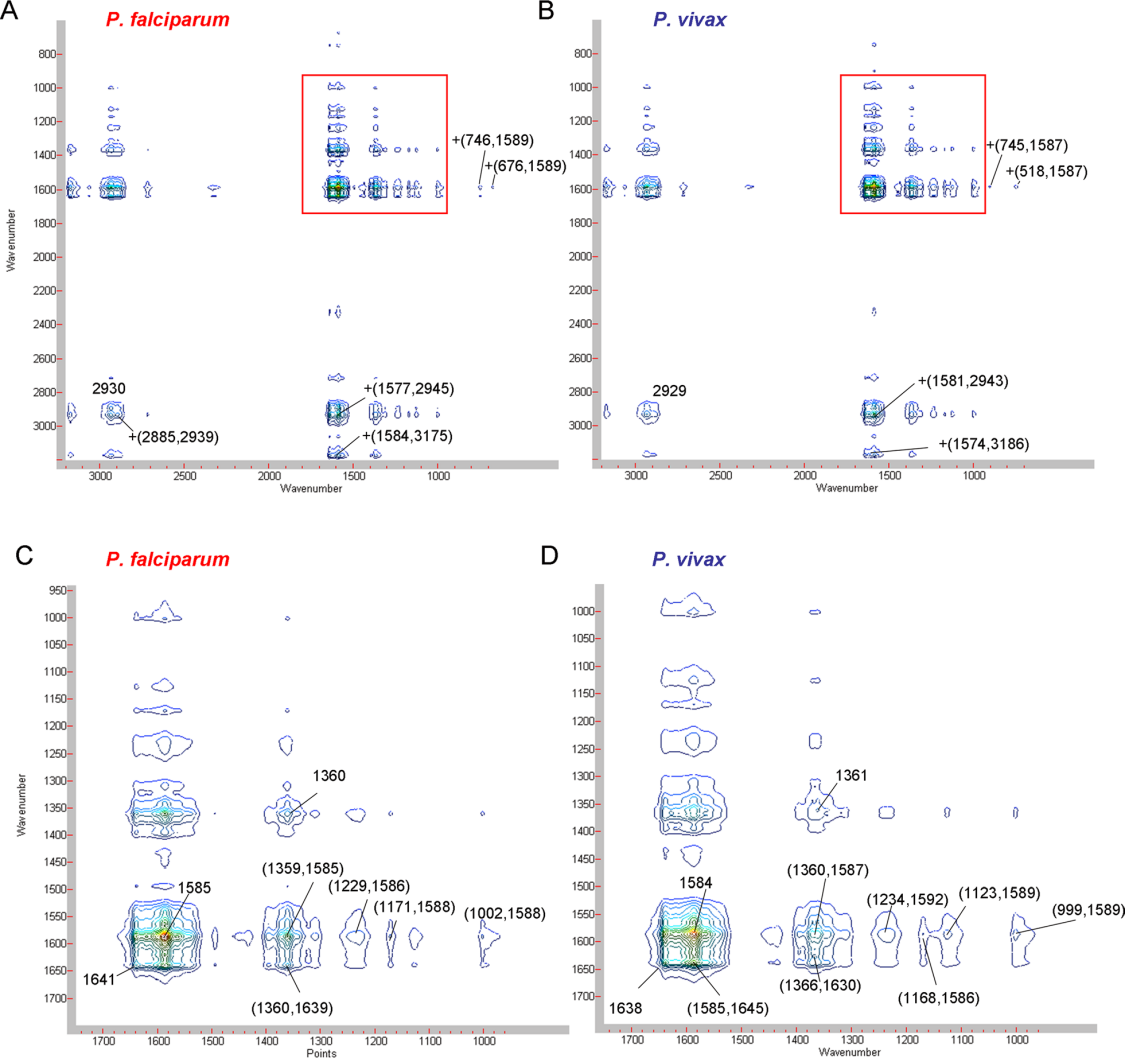
Synchronous 2D correlation of Raman spectra of malaria-infected erythrocytes on the first, fifth and tenth day of hospitalization. **A** *P. falciparum*, and **B** *P. vivax*, in the wavenumber range of 3200–600 cm^−1^; **C** *P. falciparum*, and **D** *P. vivax*, in the wavenumber range of 1750–900 cm^−1^, *vide* (**A**), and (**B**) excitation of 514.5 nm

Peaks in the synchronous correlation spectrum indicates the susceptibility of simultaneous or coincidental spectral intensity response of the selectively excited various chemical moieties of the system. In this work in effect gives you the susceptibility of spectral intensity changes reflecting various molecular moieties along the time course of infection. The synchronous 2D correlation, conducted for the Raman spectra obtained during the course of hospitalization due to infection with two types of *Plasmodium,* showed almost identical, relationships, which can be seen in the 2D spectra in Fig. 3A and B, covering the range of 3200–600 cm^−1^. The autopeaks show the greatest variability of spectral intensity in the so-called dynamic spectra; they were collected with assignments, shown in Tables 2 and 3.

**Table 3:** The prominent synchronous correlation cross-peaks for *P. falciparum* infected RBCs and their assignments, 514.5 nm laser line [8–10, 15, 22, 27, 34–37, 41]

Synchronous			Asynchronous		
Auto-peaks	Assignment		Assignment	Cross-peaks	Assignment
2930	ν_s_CH_3_; proteins and lipids; PC, outer monolayer		ν_s_CH_3_; proteins and lipids; PC, outer monolayer	−(2929,3173)	2 × ν_37_ (oxyHb)
1641	ν_10_; Hz; amide I (conf-α)		ν_10_; Hz; amide I (conf-α);	−(1640,2889)	ν_as_CH_2_; PC, outer monolayer
1585	ν_37;_ haem; His, νC_4_ = C_5_ (N_π_-H), δNH; Asp, ν_a_(COO^−^)		His, νC_4_ = C_5_ (N_π_-H), (N_τ_-H); Arg, ν_s_(CN_3_H_5_^+^); Lys, δ_as_(NH_3_^+^)	−(1633,3183)	2 × ν_37_ (oxyHb)
1360	ν_4;_ haem (deoxy); Trp, νN_1_ = C_8_ (νW7); Asp, γ(CH_2_)		ν_37_; haem (deoxy; His, νC_4_ = C_5_ (N_π_-H)	+(1581,2946)	ν_s_(CH_3_), PE, internal monolayer
			ν_4_; haem (deoxy);	+(1356,2943)	ν_s_(CH_3_), PE, internal monolayer
Assignment	Cross-peaks	Assignment	ν_10_; Hz; amide I (conf-α);	+(1642,1651)	amide I (disordered)
ν_as_CH_2_; PC, outer monolayer	+ (2885,2939)	ν_s_(CH_3_), PE, internal monolayer	ν_19_; haem; Tyr, ν(CC)	−(1609,1642)	ν_10_; Hz; amide I (conf-α);
ν_37_; haem (oxy); His, νC_4_ = C_5_ (N_π_-H), δNH;	+ (1584,3175)	2 × ν_37_ (oxyHb)	ν_2;_ Hz; _,_ Asp, ν_as_(COO^-^); His, νC_4_ = C_5_ (N_τ_-H)	−(1573,1642)	ν_10_; Hz; amide I (conf-α);
His, νC_4_ = C_5_ (N_τ_-H)	+ (1577,2945)	νs(CH3), PE, internal monolayer	Lys, δ_s_(NH_3_^+^); His, νC_4_ = C_5_ (N_π_-, N_τ_-);	−(1530,1642)	ν_10_; Hz; amide I (conf-α);
ν_4_; haem (deoxy); Trp νN_1_ = C_8_ (W7)	+ (1359,1585)	ν_37_; haem (oxy); His, νC_4_ = C_5_ (N_π_-H), δNH;	Tyr, ν(CC)	−(1519,1642)	ν_10_; Hz ; amide I (conf-α);
ν_4_; haem (deoxy); Trp, νN_1_ = C_8_ (W7); Ala	+ (1360,1639)	ν_10_; Hz;	ν_4_; Hz; Trp, νN_1_ = C_8_ (W7)	+(1371,1642)	ν_10_; Hz; amide I (conf-α);
ν_13_ or ν_42_;haem (oxy); Tyr, ν_7a’_ ν(CO)	+ (1229,1586)	ν_37_; haem (oxy); His, νC_4_ = C_5_ (N_π_-H), δNH;	ν_4_; haem (deoxy); Trp, νN_1_ = C_8_ (W7); Ala	+(1360,1642)	ν_10_; Hz; amide I (conf-α);
ν_30_; haem (deoxy); Asp; Tyr; ν_9a_,(CH); δ(COH)	+ (1171,1588)	ν_37_; haem (oxy); His, νC_4_ = C_5_ (N_π_-H), δNH;	ν_4_; haem (deoxy); Trp, νN_1_ = C_8_ (W7); Ala	−(1359,1573)	ν_2_; Hz; His, νC_4_ = C_5_ (N_τ_-H); Asp; ν_as(COO-)_
ν_47_; haem (oxy); Phe,(CC)	+ (1002,1588)	ν_37_; haem (oxy); His, νC_4_ = C_5_ (N_π_-H), δNH;	ν_4_; haem (deoxy); Trp, νN_1_ = C_8_ (W7); Ala	−(1359,1535)	Amide II; Lys, νCN, δ(NH)
Thr, γ_rock_(CH_2_); Ser; Val;	+ (746,1589)	ν_37_; haem (oxy); His, νC_4_ = C_5_ (N_π_-H), δNH;	ν_4_; haem (deoxy); Trp, νN_1_ = C_8_ (W7); Ala	+(1359,1374)	ν_4;_ haem (oxy); Asp
ν_4_; haem (oxy); Cys,(CS)	+ (676,1587)	ν_37_; haem (oxy); His, νC_4_ = C_5_ (N_π_-H), δNH;	ν_4_; haem (deoxy);	+ (671,1642)	ν_10;_ Hz; amide I (conf-α);
			ν_7_; haem (oxy); Cys,(CS)	+ (676,1361)	ν_4;_ haem (deoxy); Trp, νN_1_ = C_8_ (W7)

*Hz* haemozoin; *Ala* Alanine, *Asp* Aspartic acid, *Arg* Arginine, *Cys* Cysteine, *Gln* Glutamine, *His* Histidine, *Lys* Lysine, *Phe* Phenylalanine, *Ser* Serine, *Trp* Tryptophan, *Thr* Threonine, *Tyr* Tyrosine, *Val* Valine, *PC* phosphatidylcholine, *PE* phosphatidylethanolamine, *RC* random coil

An intense autopeak was observed for both types of *Plasmodium* for the band around 2930 cm^−1^, which indicates the importance of _s_CH_3_ vibration, including membrane lipids, in particular, phosphatidylcholine (PC) [20]. PC is a component of the outer part of the erythrocyte lipid bilayer, which is significantly altered by parasite attack [21]. Further intense autopeaks appearing for the wavenumbers 1641, 1585, 1360 cm^−1^ (*P. falciparum*) and 1638, 1585, 1361 cm^−1^ (*P. vivax)* can be mainly attributed to haem marker vibrations and also to haemozoin [15, 22]*.* However, in addition, the vibrations at approximately 1640 cm^−1^ are also due to the helical conformation of amide I [23]. It seems that domains consisting of bundles of helices or mixed helix-sheet structures of parasitic proteins, participating in the invasion of the parasite into the cell, also make a significant contribution to this peak [24–26]. Moreover, the strong aspartic acid (Asp) band assigned to the ionized carboxyl groups adds its contribution to the 1584 cm^−1^ autopeak [27]. One cannot exclude the participation of histidine (His) νC4 = C5 stretching vibrations in this peak, which occurs across a wide range of 1573–1590 cm^−1^, indicating the number of protonated nitrogen atoms and metal ion bonding [28]. Another autopeak in the vicinity of 1360 cm^−1^ is also generated by tryptophan (Trp) residues and indicates the importance of this amino acid in the action of the parasite on red blood cells. Trp is characterized by a spectroscopic doublet of 1360/1340 cm^−1^ Raman bands (the so-called W7). The dominance of the 1360 cm^−1^ band of the W7 doublet indicates the hydrophobicity of the environment in which the Trp amino acid residue is located [29].

A comparison of the two synchronous 2D correlation maps, shown in Fig. 3, revealed slight relative differences in positions and intensities between the cross-peaks of cells infected with *P. falciparum* and *P. vivax*. All synchronous cross peaks, regardless of *Plasmodium* species, are positive, see Fig. 3A, B, C and D.

**Fig. 4:**
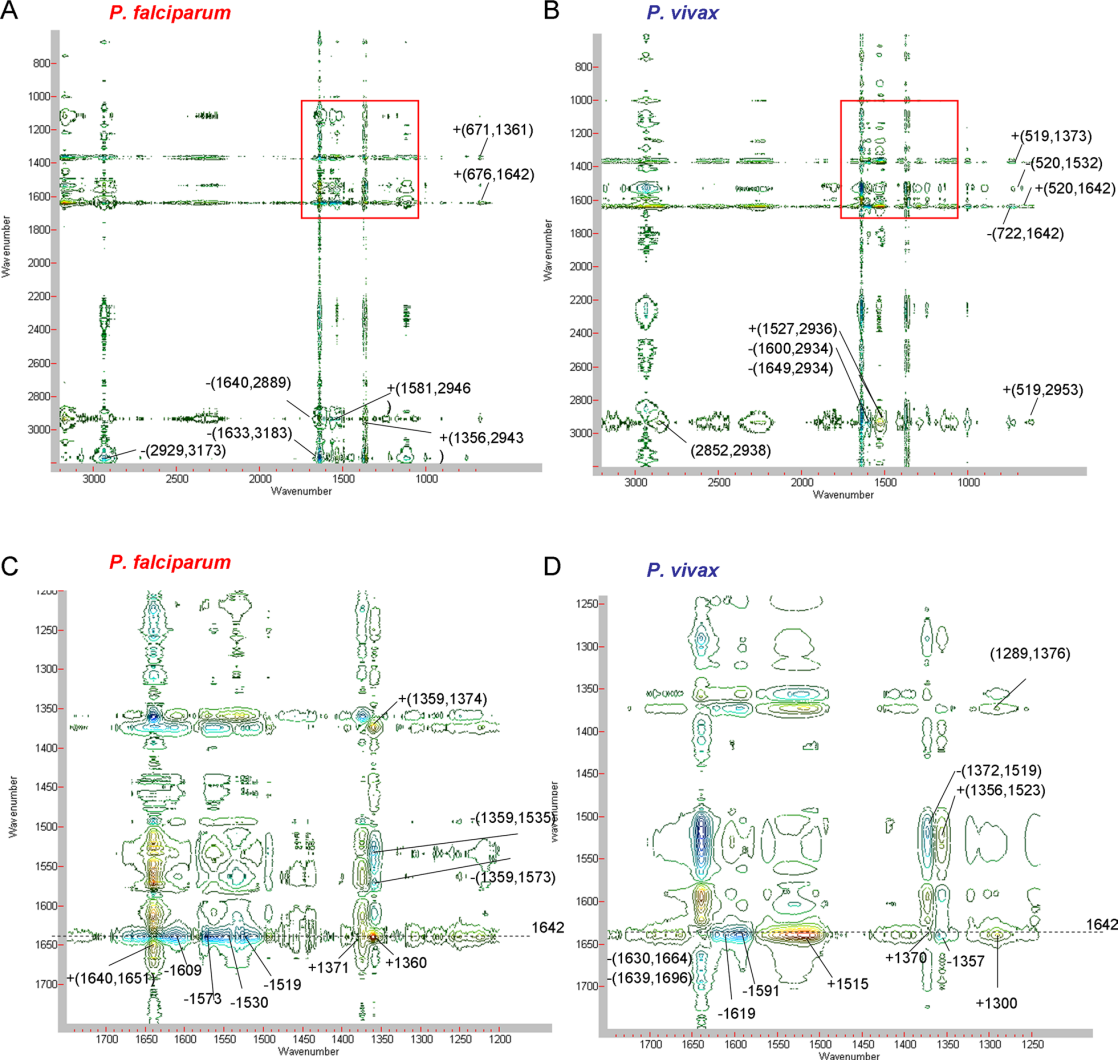
Asynchronous 2D correlation of Raman spectra of malaria-infected erythrocytes on the first, fifth, and tenth day of hospitalization. **A** *P. falciparum* and **B** *P. vivax*, in the wavenumber range of 3200–600 cm^−1^; **C** *P. falciparum*, and **D** *P. vivax*, in the wavenumber range of 1750–900 cm^−1^, *vide* (**A**) and (**B**) excitation of 514.5 nm

The intense synchronous cross-peaks for *P. falciparum* come from 2885 cm^−1^ (ν_as_CH_2_, PC) and 1577 cm^−1^ (from νC = C of His vibration) both correlates with phosphatidylethanolamine (PE) ν_s_CH_3_ vibration (approximately 2940 cm^−1^) (see Fig. 3A, and Table 3). The appearance of PC and PE derived cross-peaks in infected blood cells may be related to the developmental stages of the parasite life cycle. The greatest fluidization of the erythrocyte membrane domains, occupied by PC and PE, occurs at the beginning of the infection between the ring stage and the trophozoite stage of the parasite [30]. Vibrations of the Trp and His residues generate a series of synchronous cross-peaks in the spectra of infected blood cells, especially among cells infected with *P. falciparum*, see Table 3. This set of cross-peaks indicates the appearance of the HRP2 protein (histidine-rich protein 2) in the infected cell, which is a soluble protein in the medium, bound to the erythrocyte membrane and secreted quickly, two hours after invasion [31, 32]. Other intense synchronous cross-peaks are also generated by His νC = C vibrations (around 1590 cm^−1^) and haem vibrations, ν_13_ or ν_42_ (1229 cm^−1^), ν_30_ (1171 cm^−1^) and ν_47_ (1002 cm^−1^). A relatively low intensity, positive cross-peak at (676,1587) indicates the correlation of the νCS vibrations (of cysteine, Cys) with the ν_37_ haem vibrations [33].

Intense synchronous cross-peaks for red blood cells infected with *P. vivax* arise from the correlation of vibrations ν_s_CH_3_ (2943 cm^−1^) typical of lipids in the internal membrane layer, and νC = C from His (1581 cm^−1^), see Table 4. There are also several synchronous cross-peaks that correlate the vibrations of Trp (W7, at c.a.1360 cm^−1^) with the vibration of the haem, ν_37_ and with His νC_4_ = C_5_ (1587 cm^−1^). The rocking vibrations of the NH_3_, τ(NH_3_) groups in threonine (Thr), with a wavenumber of around 1120 cm^−1^, generate an asynchronous peak with the ν_37_ of the haem vibrations in the case of *P. vivax* [34]. The other synchronous cross peaks correlate with the vibrations characteristic of the haem, such as ν_37_ and ν_10_, ν_30_ and ν_37_, ν_22_ and ν_37_ and His. Finally, positive cross-peaks correlate the vibrations of 518 cm^−1^ skeletal vibrations of Cys SS and the 745 cm^−1^ vibrations of Thr, valine (Val) and serine (Ser) with the vibrations ν_37_ (1587 cm^−1^) of haem and His [33, 35–37].

In summary, synchronous cross-peaks reflect the activity of the parasite, which produces and then exports many of its proteins, the most important being PfHRP2 and PvRBP, for *P. falciparum* and *P. vivax*, respectively, to the erythrocytes cytosol and its membrane [38]. Therefore, synchronous cross-peaks characterize the parasite-haem connections inside the blood cell during protein export to the cell membrane [32, 39]. Participation in the creation of cross-peaks by haemozoin and amino acids, mainly His, indicates the function of parasite proteins, e.g., PfHEP2. *Plasmodium-* derived PfHEP2 protein, which is involved in haem detoxification, contains 60–70% His and alanine (Ala) residues. When the parasite transforms into the trophozoite stage, it captures around 40% of the blood cell volume. The host cell surface becomes covered with small knobs in which there is a parasite knob-associated, histidine-rich protein (KAHRP) or Trp-rich PfEMP1 (*P. falciparum* erythrocyte membrane protein 1) and the surface becomes parasitic in origin [24, 40].

### 2D asynchronous correlation

The signs of asynchronous cross peaks provides the sequential order of intensity changes of different constituents. Therefore, on asynchronous maps, attention is drawn to a group of asynchronous cross-peaks that appear within a similar range of wave numbers but have the opposite sign, indicating a different sequence of events influencing their generation, see Fig. 4A and B. Identifying just some of the intense cross peaks for *P. falciparum,* there are cross-peaks that correlate the vibrations of the membrane lipids, in which changes in the inner membrane layer (PE, at c.a 2945 cm^−1^) are later than that in haem ν_4_ (at 1356 cm^−1^) and ν_37_ (at 1581 cm^−1^), see Table 3. A series of negative cross peaks correlates the ν_10_ vibration at 1642 cm^−1^ (corresponding to changes in haemozoin, as well as in the secondary structure of helical proteins, also in parasitic proteins) and are ahead of the changes represented by the ν_19_ haem vibrations, together with tyrosine (Tyr) (1609 cm^−1^), His (1573 cm^−1^) and lysine (Lys) (1530 cm^−1^) [22, 24, 25, 28, 41]. The vibrations of ν_4_ in the deoxy-structure and that of Trp residue (1360 cm^−1^) correlates positively with the ν_10_ of haemozoin (1642 cm^−1^) [22, 28]. On the other hand, the vibration of ν_4_ in the deoxy-structure (1359 cm^−1^), also coinciding with the Trp position, correlates asynchronously and negatively with the ν_4_ vibration characterizing the oxidized haem structure (1374 cm^−1^) [15, 28]. Interesting are two negative cross-peaks, generated by the ν_4_ vibration of the haem deoxy- structure, (−(1359,1573)) and (−(1359,1535)). The first is derived from the ν_4_ of haemozoin with Asp and His (1573 cm^−1^), while the N–H bending vibration in the plane and the C–N stretching vibration for Lys (1535 cm^−1^) generates a second asynchronous peak for *P. falciparum* [28, 41].

There are also many asynchronous cross-peaks observed in red blood cells infected with *P. vivax;* it is worth mentioning a few intense cross-peaks generated by the vibrations of membrane lipids, see Fig. 4B and D. The peak generated by the ν_s_CH_2_ (PC) vibrations (2852 cm^−1^) correlates asynchronously with v_s_CH_3_ (PE) (2938 cm^−1^) [23]. Another positive cross-peak correlates the vibrations of Lys(1527 cm^−1^) with the vibrations of PE (2936 cm^−1^) belonging to the inner monolayer, see Table 4 [23, 41]. The next two negative peaks, the first correlating His (1630 cm^−1^) with amide I of conf-β (1664 cm^−1^) and the second cross-peak correlating haemozoin ν_10_ vibration (1639 cm^−1^) with amide I of the antiparallel β-sheet conformation (1696 cm^−1^) signify intense membrane modification through possible invasion pathway formation [42]. The band at 1696 cm^−1^, indicating the νC = O stretching vibrations of non-hydrated C = O groups of Asp residue, is a sign of aspartic proteinase activity [27, 43]. The hydrophobicity of the environment in which the Trp residue is located, is confirmed by the intense indole band for the first overtone νW18 at ca. 1515 cm^−1^, which is preceded by changes in haemozoin v_10_ [22, 28]. The presence of this positive asynchronous cross-peak illustrates the changes taking place in the host receptor under the influence of interaction with the parasite proteins (of amide I conf- α) and with the haem detoxification product, haemozoin [40].

There are two pairs of negative cross-peaks that are derived from vibrations of molecular groups that differ slightly, for *P. falciparum* at –(1609,1642) and –(1573,1642), while for *P. vivax* at –(1619,1642) and –(1591,1642), see Tables 3 and 4. These cross-peaks are generated by the 1642 cm^−1^ of the haemozoin v_10_ and peptide bond vibrations of conf-α, derived from the helical structures of the *Plasmodium* proteins, i.e., PfRH5 and PvRBP2, see Tables 3 and 4 [44, 45]. These vibrations precede subsequent events that are connected and correlate the moieties of characteristic amino acids, forming the respective ligand-receptor binding domains, i.e., Tyr and Asp and/or His in case of *P. falciparum,* and Tyr and/or Trp and Tyr and/or His for *P. vivax* [23, 28, 36]. Parasite ligand variation in expression makes analysis and diagnosis difficult [46].

**Table 4:** The prominent synchronous correlation cross-peaks for *P. vivax* infected RBCs and their assignments, 514.5 nm laser line [8–10, 15, 22, 27, 34–37, 41]

Synchronous			Asynchronous		
Auto-peaks	Assignment		Assignment	Cross-peaks	Assignment
2929	ν_s_(CH_3_); proteins and lipids, PC, outer monolayer_3_		ν_s_(CH_2_); proteins and lipids; PC, outer monolayer	+ (2852,2938)	ν_s_(CH_3_), PE, internal monolayer
1638	ν_10; Hz_		amide I (conf-α)	−(1649,2934)	ν_s_(CH_3_), PC, outer monolayer
1584	ν_37_ ; haem; Hz; His, νC_4_ = C_5_(N_π_-H); Asp		ν_19;_ haem (oxy)	−(1600,2934)	ν_s_(CH_3_), PC, outer monolayer
1361	ν_4;_ haem (deoxy); Trp, νN_1_ = C_8_ (W7); Asp, γ(CH_2_)		Lys, δ_s_(NH_3_^+^); His, νC_4_ = C_5_ (N_π_-, N_τ_-);	+ (1527,2936)	ν_s_(CH_3_), PE, internal monolayer
			Cys, ν(SS)	+(519,2953)	ν_as_(CH_3_), out-of-plane, PC, outer monolayer
Assignment	Cross-peaks	Assignment	His, νC_4_ = C_5_ (N_π_-H), (N_τ_-H)	−(1630,1664)	Hz; amide I (conf-β);
ν_as_(COO^-^), Asp; His, νC_4_ = C_5_ (N_τ_-H)	+ (1574,3186)	2 × ν_37_ (oxyHb)	ν_10_; Hz; amide I (conf-α);	−(1639,1696), w	amide I (antiparallel β-sheet),
ν_37_ ; haem (deoxy)_;_ His νC_4_ = C_5_ (N_π_-H), δNH;	+ (1581,2943)	ν_s_(CH_3_), PE, internal monolayer	ν(CC), Tyr;	−(1619,1642)	ν_10_; Hz; amide I (conf-α);
ν_37_; haem; Asp; His, νC_4_ = C_5_ (N_π_-H), δNH;	+ (1585,1645)	ν_10_; Hz; amide I (conf-α);	ν_(CC),_ Tyr; His, νC_4_ = C_5_ (N_π_-H), δNH;	−(1591,1642)	ν_10; Hz_; amide I (conf-α);
ν_4_; haem; Trp νN_1_ = C_8_ (W7)	+ (1366,1630)	His, νC_4_ = C_5_ (N_π_-H, N_τ_-H), δNH	ν(C=C), Tyr; Trp (2 × W18)	+(1515,1642)	ν_10_; Hz; amide I (conf-α);
ν_4_; haem (deoxy); Trp, νN_1_ = C_8_ (W7)	+ (1360,1587)	ν_37_; haem; His, νC_4_ = C_5_ (N_π_-H), δNH	ν_4;_ haem (deoxy); Trp νN_1_ = C_8_ (W7)	−(1357,1642)	ν_10_; Hz; amide I (conf-α);
ν(CO); Tyr, ν_7a’_ ν(CO)	+ (1234,1592)	ν(C=C), Gln, δ(NH_2_); Trp, W2	ν_21_; haem; Ala, δ(CN)	+(1300,1642)	ν_10_; Hz; amide I (conf-α);
ν_30_; haem (deoxy); Tyr, ν_9a_,(CH); δ(COH)	+ (1168,1586)	ν_37_; haem (oxy); His, νC_4_ = C_5_ (N_π_-H), δ(NH)	Thr, ω(COO^−^)	−(722,1642)	ν_10;_ Hz; amide I (conf-α);
ν_22_; haem (deoxy); Asp, Glu, ν(CO); Thr, τ(NH_3_)	+ (1123,1589)	ν_37_; haem (oxy); His, νC_4_ = C_5_ (N_π_-H), δNH	Cys, ν(SS)	+(520,1642)	ν_10_; Hz; amide I (conf-α);
ν_47_; haem (deoxy); Phe, ν(CC)	+ (999,1589)	ν(CC); Tyr; His, νC_4_ = C_5_ (N_π_-H), δNH	ν_4;_ haem (oxy); Trp, νN_1_ = C_8_ (W7)	−(1372,1519)	ν(C=C), Tyr-OH; Trp (2 × W18)
Thr, τ(COH); Trp (W18); Val	+ (745,1587)	ν_37_; haem (oxy); His, νC_4_ = C_5_ (N_π_-H), δ(NH)	ν_4;_ haem (deoxy); Trp νN_1_ = C_8_ (W7)	+(1356,1523)	δ_s_(NH_3_^+^), Lys
Cys, ν(SS)	+ (518,1587)	ν_37_; haem (oxy); His, νC_4_ = C_5_ (N_π_-H), δ(NH)	Cys, ν(SS)	−(515,1532)	amide II; Lys, δ_s_(NH_3_^+^); His, νC_4_ = C_5_ (N_π_-, N_τ_-);
			ν_21_; haem; Ala, δCN	+(1289,1376)	Lys, δ_s_(NH_3_^+^); His, νC_4_ = C_5_ (N_π_-, N_τ_-)
			Cys, ν(SS)	+(519,1373)	Lys, δ_s_(NH_3_^+^); His, νC_4_ = C_5_ (N_π_-, N_τ_-)

*Hz* haemozoin; *Ala* Alanine, *Asp* Aspartic acid, *Arg* Arginine, *Cys* Cysteine, *Glu* Glutamine, *His* Histidine, *Lys* Lysine, *Phe* Phenylalanine, *Trp* Tryptophan, *Thr* Threonine, *Tyr* Tyrosine, *Val* Valine, *PC* phosphatidylcholine, *PE* phosphatidylethanolamine:

The reverse sequence of events for both types of *Plasmodium* is clearly visible on the asynchronous maps, see Fig. 4. In relation to *P. falciparum,* a negative asynchronous cross-peak appears −(1530,1642), while in the case of *P. vivax*, this is positive +(1515,1642). Regarding the first *P. falciparum* cross-peak, changes occur in amide I conf-α and in haemozoin, which correlate with the subsequent changes in Lys and His residues [23, 28, 41]. In the case of the second *P. vivax* positive cross-peak, the changes begin with Tyr and/or Trp that cause the rearrangement of amide I conf- α vibrations [28, 36].

The second pair of intriguing cross-peaks with opposite signs indicates: positive +(1360,1642) for *P. falciparum* and negative –(1357,1642) for *P. vivax*. Vibrations with a wave number of 1360 cm^−1^ for *P. falciparum* are caused due to changes in Trp, proving that this takes place in a hydrophobic environment [28]. As regards the second cross-peak, the 1357 cm^−1^ vibrations, assigned to ν_4_ haem, follow signals from the haemozoin and/or parasite protein secondary structure vibrations [22]. The difference in the position of the Trp band, observed in erythrocytes infected with different types of parasites, indicates a different positioning of this amino acid residue relative to the haem [28]. This, in turn, indicates a different pathway of vibrational energy flow for both parasite infections [47]. It can be concluded that the dynamics observed in these cross-peaks represent the initial invasion stage in both these cases [39].

These two pairs of intense asynchronous cross-peaks seem very indicative. They reflect the process and specificity of the formation of the transmembrane complex of the parasite ligand and the host cell receptor. PfRH5 consists of two helical bundles and some amino acid residues (mostly His) that are aligned with the basigin receptor binding site [44]. Basigin has evolved two ways of binding. In trans-recognition, basigin attaches soluble protein or protein on an adjacent cell. In cis-recognition, basigin binds to proteins in the same cell, especially in the same membrane [48]. The presence of the conf. α-rich proteins of the parasite is indicated by a vibration at position 1642 cm^−1^, which together with the vibration of Trp residues around 1360 cm^−1^ indicates the hydrophobic nature of pockets on the basigin, generating a positive asynchronous correlation peak (see Table 3) [49, 50]. Amino acids such as His, Trp, Tyr, and Lys, which generate cross peaks for *P. falciparum*, indicate their involvement in the formation of the PfRh5-basigin complex [49].

The corresponding complex for *P. vivax* invasion uses transferrin receptor (TfR) as the host receptor, as indicated by the higher rates of parasite invasion into young reticulocytes, which have a high level of TfR [39]. This is consistent with the observed cross-peaks from the amino acids, Asp, Tyr and His, characteristic of the iron binding site (see Table 4) [51]. It is worth noting that on 2D spectra for the *P. vivax* infection, there are asynchronous cross-peaks in which νSS vibrations from Cys residues are involved (see Table 4) [33]. This indicates that PvRBPs, which are rich in cysteine residues, build up important interactions [26]. In this context, peak +(519,2953) indicates the effect of PvRBP Cys- rich domains on changes in the host’s biological membrane (see Table 3) [30].

Involvement in gametocyte formation after early reticulocyte invasion has also been reported, which may be an additional reason for the generation of the observed differences in the sequence of events in the 2D correlation spectra [39]. The full development of *P. falciparum* gametocytes takes place simultaneously in the erythrocyte phase of the parasite's life cycle and proceeds through a series of intermediate stages, lasting up to 9–12 days. [52]. A factor that may contribute to the initiation of this process may be, for example, contact with anti-malarial drugs that are used in hospital conditions. Such external stimuli can lead to an increased tendency to produce gametocytes [6].

### Whole blood EPR spectroscopy

**Fig. 5:**
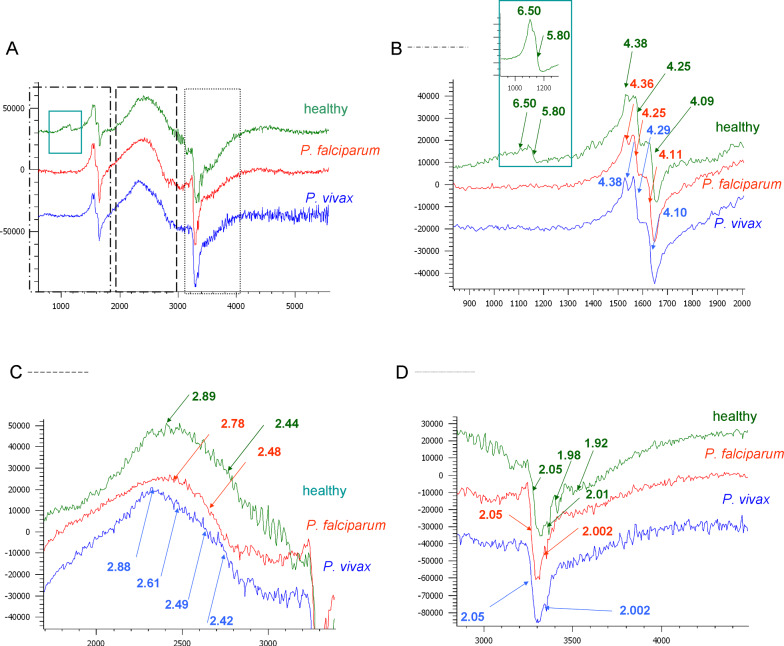
EPR spectra of human whole blood measured in the X-band of: **A** healthy blood (upper spectrum), *P. falciparum*-infected blood (middle spectrum) and *P. vivax* infected blood (lower spectrum) both at the beginning of hospitalization, in the magnetic field range of 600–5600 Gs; **B** 900–2000 Gs; **C** 2000–3500 Gs; **D** 3000–5000 Gs

The paramagnetic centers, the signal of which can be expected in human blood, are compounds of iron and copper and also free radicals (see Table 5) [53, 54]. EPR spectra depend on the local symmetry in which the paramagnetic center is located, as well as on the oxidation and spin state, therefore, they provide information regarding the surroundings of the ion with paramagnetic properties [55]. Hence, the task was to verify which changes occur in the blood, due to the activity of the malaria parasite. The EPR spectra of the healthy blood (upper spectrum), *P. falciparum*-infected blood (middle spectrum) and *P. vivax-*infected (lower spectrum) at the beginning of hospitalization are presented in Fig. 5A. Spectra in the narrower ranges of magnetic fields are shown in Fig. 5B–D, respectively. The range of resonant fields from 900 to 2000 Gs includes the characteristic signals from ferric haem, while the ferrous haem is undetectable (see Fig. 5B). Ferric compounds of a high-spin of 5/2 are characterized by a distinctive g_⊥_- factor of around 6.0. The third g-factor component of this methaemoglobin signal appears around 2 and is hidden under the other visible signals in this region, see Fig. 5D [56]. The fragment of the EPR spectrum from healthy blood, shown in Fig. 5B in the inset, with characteristic g-values of 6 and 5.80, is analogous to the typical signal observed for the iron of isolated alpha-haemoglobin chains [57]. This signal is not observed in the case of *Plasmodium*-infected blood, as shown in Fig. 5B.

**Table 5:** Observed EPR resonance signals and their assignments for healthy and *Plasmodium* infected human whole blood, at 78 K, X-band

Blood sample: healthy/infected	Observed g-factors	Assignment	References
Healthy	g = 6.50, 5.80, g = 1.98	High-spin ferrihaem centers, 3d^5^ signal from blood	[53, 54, 56, 57]
Healthy	g = 4.38, 4.25, 4.09 (4.10)	High-spin ferric centers non-haem proteins (e.g. transferrin) signal from plasma	[54, 55, 59]
*P. falciparum*	g = 4.36, 4.25, 4.11;		
*P. vivax*	g = 4.38, 4.29, 4.10		
Healthy	g = 2.89, 2.44;	High-spin ferric centers non-haem proteins (e.g. polynuclear ferric aggregates, ferritin) signal from plasma?	[60]
*P. falciparum*	g = 2.78, 2.48;		
*P. vivax*	g = 2.88, 2.61, 2.49, 2.42		
healthy	g_⊥_ hidden (≈2.20), g = 1.92	Low spin ferrihaem centers; axial type III, 3(d_*xz*_,d_*yz*_)^4^(d_*xy*_)^1^ ground state (eg. bis-histydyl binding to haem) signal from blood	[59, 61]
Healthy *P. falciparum P. vivax*	g_⊥_ = 2.05, g hidden (≈ 2.18)	Type 2 Cu^2+^ signal from plasma	[62]
healthy	g = 2.01;	Peroxyl radical; signal from blood	[63, 65, 66] [67–69]
*P. falciparum*	g = 2.002;	Tyrosyl radical signal from blood	
*P. vivax*	g = 2.002

In the region of the g-factor 4.10–4.40, a characteristic signal is visible, observed for Fe^3 +^ in non-haem proteins [58]. These signals, indicating paramagnetic centers of rhombic symmetry for normal blood and blood infected with *P. falciparum* and *P. vivax* do not differ from one other, see Fig. 5C. These anisotropic signals can be attributed to the high-spin ferric ions found in transferrin.

Regarding healthy as well as infected blood, a very wide band develops in the next range of magnetic fields, for g = 2.30–2.90, see Fig. 5C. This range is characterized by low-spin, ferri-haem centres [59]. The characteristic broadband profile at 78 K indicates that it may be composed of phases typical of iron storage proteins [60]. Therefore, signals of this type have been classified, among others, as iron aggregates, possibly in ferritin. The maximum of this broad signal has clearly shifted in the case of the infected blood compared to healthy blood, and as regards the infected blood, the maximum depends on the type of parasite, see Fig. 5C. The maximum band for blood infected with *P. falciparum* is g = 2.78, and for *P. vivax* is g = 2.88. This broad band probably comes from overlapping low- and high-spin iron centers with different local symmetries [56, 61].

Figure 5D shows the signal g = 1.92 for EPR spectra of healthy blood, typical of low-spin, ferri-haem complexes [59]. Basically, this spectral range describes copper centers with tetragonal symmetry, as indicated by the characteristic coefficient, g = 2.05 [62].

In this range, narrow signals around 3000 Gs are also clearly visible. Biological EPR signals with g-factors close to 2 are usually interpreted as free radicals [63] and peroxyl radicals are generated by hydrogen peroxide in haem proteins [64]. The signal with g = 2.01, observed in healthy blood, can be attributed to this radical [65, 66]. The second radical signal with g = 2.002 in the infected blood comes from the tyrosyl radical. It was found that the g-factor characterizing the tyrosyl radical is sensitive to local charge densities, hence the differences in radical formation should match the different protein structures [67–69].

Pathologies are an additional factor influencing the formation of radicals [64, 70]. In fact, human red blood cells, infected with the trophozoite, *P. falciparum,* produce around twice as many H_2_O_2_ and OH radicals than normal erythrocytes. This characteristic was not observed at the ring stage when digestion of the host cells had not yet begun. Therefore, it is believed that reactive oxygen species are produced in the parasite's food vacuole during the digestion of the host cell's cytosol and, therefore, remain in the host cell [71].

### 2T2D correlation of whole blood infected with *Plasmodium*

**Fig. 6:**
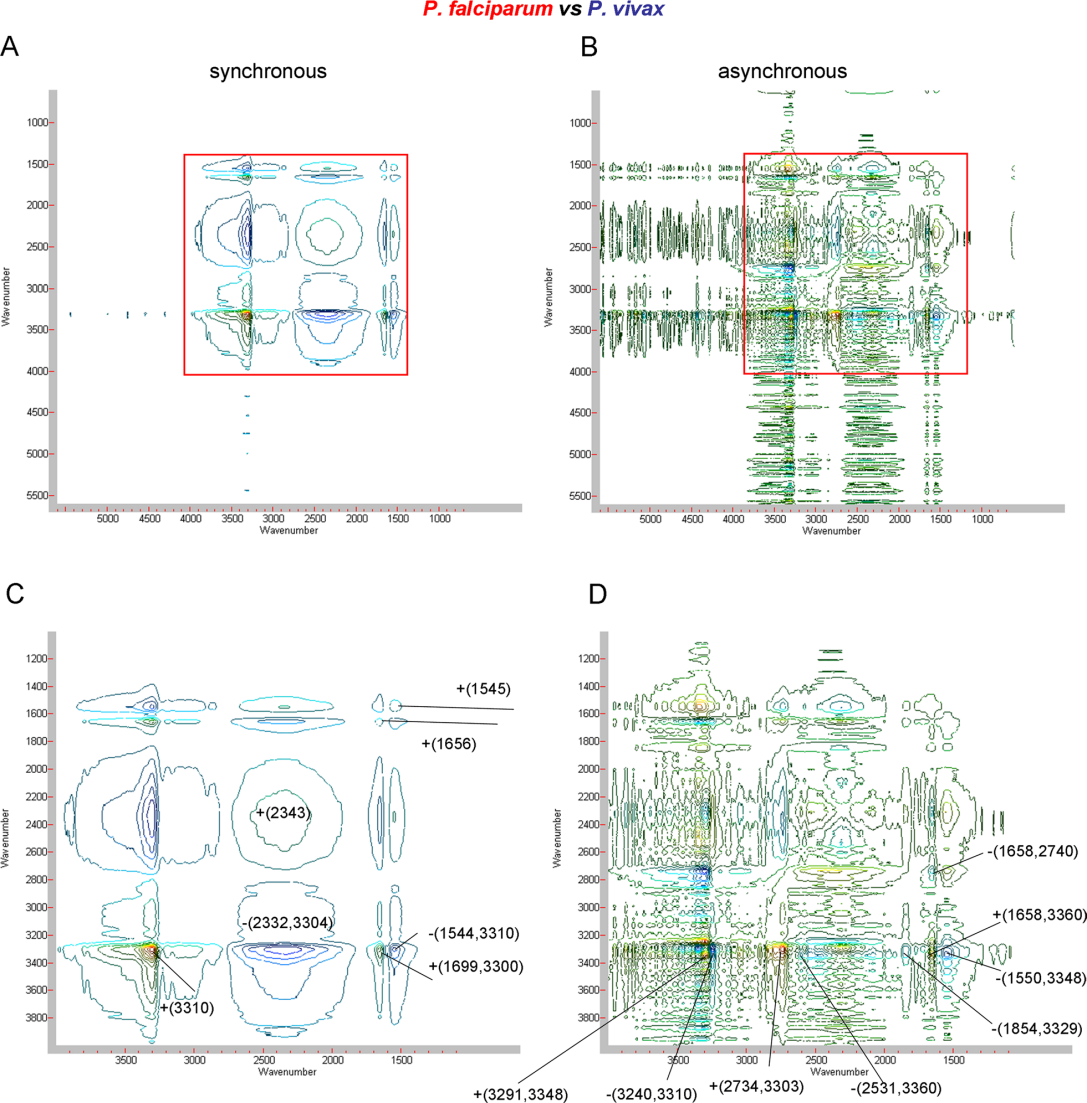
Synchronous (**A**) and asynchronous (**B**) 2T2D correlation EPR spectra of human whole blood infected with *P. falciparum vs P. vivax* in the magnetic field range of 5600–600 Gs; synchronous (**C**) and asynchronous (**D**) 2T2D correlation EPR spectra of human whole blood infected with *P. falciparum vs P. vivax* in the magnetic field range of 4000–1500 Gs

Two-trace two-dimensional correlation spectroscopy (2T2D), gives the possibility to compare a pair of spectra in the formalism of two-dimensional correlation as a 2D map [14]. This analysis allows preferentially to indicate similarities or differences of blood samples with respect to paramagnetic centers under the influence of different types of *Plasmodium*. The correlation of two whole blood spectra in the initial phase of the *P. falciparum* and *P. vivax* infection provide an interesting comparison, see Fig. 6 and Table 6. The synchronous spectrum shows the dominant spectral features in the two compared EPR spectra [14]. Significant correlation relationships appear in the area of 1500–4000 Gs resonance fields, marked in Fig. 6A and B. The most intense auto-peak occurs at 3310 Gs (peroxyl radical) and at 2343 Gs (high-spin Fe^3+^ polynuclear aggregates, non-haem type proteins), while this is clearly weak in the low field range for 1656 Gs and 1545 Gs (both high-spin Fe^3+^, non-haem proteins). Peaks appearing outside the diagonal positions, the so-called cross-peaks, show a similar trend of changes between the two spectral intensities. Positive cross-peak appear for +(1699,3300) due to hs Fe^3+^, non-haem centres and peroxyl radicals. Negative cross-peaks −(2332,3304) and −(1544,3310) are derived from low-spin Fe^3+^, haem and high-spin Fe^3+^, non-haem centres and peroxyl radicals (Fig. 6C).

**Table 6:** The prominent 2T2D synchronous and asynchronous EPR correlation cross-peaks and their assignments for *P. falciparum* and *P. vivax* infected human whole blood, at 78 K, X-band (expressed as the size of the resonance fields [Gs] and the g-factors)

Synchronous			Asynchronous		
Auto-peaks (g-factor)	Assignment		Assignment	Cross-peaks (g-factor)	Assignment
+ (3310) 2.025	Peroxyl radical		Cu^2+^-complexes	+ (3291,3348) + (2.037, 2.002)	Tyrosyl radical
+ (2343) 2.861	hs Fe^3+^, non-haem aggregates, linked to protein		Cu^2+^-complexes	−(3240,3310) −(2.069, 2.025)	Peroxyl radical
+ (1656) 4.048	hs Fe^3+^, non-haem, in transferrin		hs Fe^3+^, non-haem aggregates	+(2734,3303) +(2.452, 2.030)	Peroxyl radical
+ (1545) 4.340	hs Fe^3+^, non-haem, in transferrin		hs Fe^3+^, non-haem aggregates	−(2531,3360) −(2.648, 1.995)	ls Fe^3+^, haem
Assignment	Cross-peaks (g-factor)	Assignment	hs Fe^3+^, non-haem aggregates	−(2185,3329) −(3.068, 2.014)	Peroxyl radical
hs Fe^3+^, non-haem aggregates	−(2332,3304) −(2.874, 2.029)	Peroxyl radical	hs Fe^3+^, non-haem, transferin	+(1658,3360) +(4.042, 1.995)	ls Fe^3+^, haem
hs Fe^3+^, non-haem	+(1699,3300) +(3.945,2.031)	Peroxyl radical	hs Fe^3+^, non-haem, transferin	−(1550,3348) −(4.325, 2.002)	Tyrosyl radical
hs Fe^3+^, non-haem, transferin	−(1544,3310) −(4.342,2.025)	Peroxyl radical	hs Fe^3+^, non-haem, transferin	−(1658,2740) −(4.042, 2.446)	hs Fe^3+^, non-haem aggregates

*ls* low-spin, *hs* high-spin,

The 2T2D asynchronous spectrum contains cross-peaks located outside the diagonal positions (see Fig. 6B and Table 6). Two bands that correspond to the spectral coordinates of cross-peak, in the 2T2D asynchronous spectrum, come from vibrations of different moieties [14]. Two asynchronous peaks indicate that there are more copper centres in *P. falciparum* than the tyrosyl radicals(+(2.037, 2.002)) but fewer than the peroxyl radicals (−(2.069, 2.025)). The appearance of these cross-peaks indicate the importance of the multifunctional action of ceruloplasmin, affecting the changes taking place in the infected blood [72]. Several cross-peaks indicate ferric, high-spin signals, which clearly differ in the local symmetry of the surrounding proteins and characterize them. They are likely to define aggregated iron-storing, multinuclear species (+ (2.452, 2.030) with peroxyl radical, −(2.648, 1.995) with ls Fe^3+^, haem and −(3.068, 2.014) with peroxyl radical). These cross-peaks indicate that the spectral intensity contribution of ferric centers in the non-haem storage proteins of *P. falciparum* infected blood are observed in abundance. Regarding the *P. falciparum* infection, more low-spin iron haem centres appear than high-spin sites, associated with non-haem proteins (+(4.042, 1.995)), thereby indicating the virulence of this parasite. Another cross-peak (−(4.325, 2.002)) indicates that tyrosyl radicals are also generated in abundance in this infection. On the analysed map, the cross-peak (−(4.042, 2.446)) shows that more high-spin iron centres are observed in non-haem proteins related to iron transport, e.g., ferritin, than in iron accumulating proteins. The observed cross-peaks indicate that there is a specificity of the iron recycling rate in the blood for each type of parasite and identify which paramagnetic centers are important in this process.

## Conclusions

Raman spectroscopy was used to monitor changes in the blood cells of patients diagnosed with malaria, who were treated in the University Hospital in Krakow, Poland. Statistical analysis showed no significant differences in standard laboratory tests between the two groups of patients diagnosed with *P. falciparum* or *P. vivax*.

The Raman spectra of red blood cells infected with *Plasmodium*, at the same time of hospitalization, were quite similar; it was difficult to identify the characteristics associated with a particular species of malaria parasite directly from the spectra.

The autopeak for wavenumber 1641 cm^−1^ observed on the 2D synchronous map for erythrocytes of patients diagnosed with *P. falciparum* malaria related to the formation of haemozoin is clearly shifted in relation to the position observed in the dynamic spectra (Table 3). This indicates the significant contribution to the 1641 cm^−1^ cross-peak of peptide bond vibrations from parasitic proteins in which numerous helical domains are present.

The most characteristic feature is the pattern of asynchronous 2D maps obtained for both types of *Plasmodium*, indicating a different dynamics of activity of both types of parasite, see Fig. 4C and D. An example of a difference in dynamics in the sequence of events is the opposite sign of the cross-peaks: positive +(1360,1642) for *P. falciparum* and negative −(1357,1642) for *P. vivax*. The first cross-peak listed for *P. falciparum* is generated by the ν_4_ deoxy-haem; Trp and Ala vibrations appears ahead of the ν_10_ and amide I (conf. α) vibrations. In this case of *P. vivax,* changes described by ν_10_ and amide I (conf. α) appear before those in ν_4_ and Trp. *Plasmodium* invasion proceeds through a series of complex stages of receptor-ligand interaction, a different pathway of vibrational energy flow, which is indicated by asynchronous cross-peaks. The observed dependencies show that these processes for each type of parasite have different time frames and are faster in the case of *P. falciparum.* Cross peaks from amino acids such as His, Trp, Tyr and Lys observed for *P. falciparum* indicate their participation in the formation of the PfRh5-basigin complex. In the case of *P. vivax* infection, asynchronous 2D cross peaks were demonstrated, in which νSS vibrations from Cys residues indicate the cysteine- rich protein PvRBP.

EPR spectra obtained at the beginning of the infection, analysed with the 2T2D method, indicate some differences in the iron recycling process, e.g., regarding its storage in proteins. In blood infected with *P. falciparum*, correlation peaks manifest the presence of numerous iron centers characterizing non-haem blood storage proteins. More low-spin iron haem centres associated with non-haem proteins are observed for *P. falciparum* infection, indicating the virulence of this parasite.

To our knowledge, this is the first observation in which *Plasmodium* spp. were discriminated using the 2D-COS method to analyse their activity through their influence on the erythrocytes of hospitalized patients, through the use of Raman and EPR spectroscopy. This analysis is intended to contribute to greater understanding of the phenomena occurring in red blood cells during infection with various types of *Plasmodium*.

## Data Availability

All data generated or analyzed during this study are included in this published article. The datasets analyzed during the current study are available from the corresponding author on reasonable request. Samples are not available.
